# Curcumin Inhibits the PERK-eIF2*α*-CHOP Pathway through Promoting SIRT1 Expression in Oxidative Stress-induced Rat Chondrocytes and Ameliorates Osteoarthritis Progression in a Rat Model

**DOI:** 10.1155/2019/8574386

**Published:** 2019-05-16

**Authors:** Kai Feng, Yuwei Ge, Zhaoxun Chen, Xiaodong Li, Zhiqing Liu, Xunlin Li, Hui Li, Tingting Tang, Fei Yang, Xiaoqing Wang

**Affiliations:** ^1^Shanghai Key Laboratory of Orthopedic Implants, Department of Orthopedics, Ninth People's Hospital, Shanghai Jiaotong University School of Medicine, Shanghai, China; ^2^Department of Bone and Joint Surgery, Renji Hospital, Shanghai Jiaotong University School of Medicine, Shanghai, China

## Abstract

Oxidative stress plays a crucial role in the occurrence and development of osteoarthritis (OA) through the activation of endoplasmic reticulum (ER) stress. Curcumin is a polyphenolic compound with significant antioxidant and anti-inflammatory activity among various diseases. To elucidate the role of curcumin in oxidative stress-induced chondrocyte apoptosis, this study investigated the effect of curcumin on ER stress-related apoptosis and its potential mechanism in oxidative stress-induced rat chondrocytes. The results of flow cytometry and terminal deoxynucleotidyl transferase-mediated dUTP nick-end labeling (TUNEL) staining showed that curcumin can significantly attenuate ER stress-associated apoptosis. Curcumin inhibited the expression of cleaved caspase3, cleaved poly (ADP-ribose) polymerase (PARP), C/EBP homologous protein (CHOP), and glucose-regulated protein78 (GRP78) and upregulated the chondroprotective protein Bcl2 in TBHP-treated chondrocytes. In addition, curcumin promoted the expression of silent information regulator factor 2-related enzyme 1 (SIRT1) and suppressed the expression of activating transcription factor 4 (ATF4), the ratio of p-PERK/PERK, p-eIF2*α*/eIF2*α*. Our anterior cruciate ligament transection (ACLT) rat OA model research demonstrated that curcumin (50 mg/kg and 150 mg/kg) ameliorated the degeneration of articular cartilage and inhibited chondrocyte apoptosis in ACLT rats in a dose-dependent manner. By applying immunohistochemical analysis, we found that curcumin enhanced the expression of SIRT1 and inhibited the expression of CHOP and cleaved caspase3 in ACLT rats. Taken together, our present findings firstly indicate that curcumin could inhibit the PERK-eIF2*α*-CHOP axis of the ER stress response through the activation of SIRT1 in tert-Butyl hydroperoxide- (TBHP-) treated rat chondrocytes and ameliorated osteoarthritis development in vivo.

## 1. Introduction

Osteoarthritis (OA) is a chronic degenerative joint disease mainly featured by degradation of articular cartilage and remodeling of the underlying bone and synovitis resulting in pain and disability [1, 2]. Osteoarthritis is one of the most common and disabling diseases in the world. It affects about 40% of people over the age of 70 and is associated with an increased risk of comorbidity and death [2].

Chondrocyte is the only cell type in an articular cartilage; common healthy chondrocyte can maintain cellular homeostasis under oxidative stress. Oxidative stress is one of the important factors that led to osteoarthritis, and superabundant oxidative stress may cause the death of chondrocyte ultimately [3]. Reactive oxygen species (ROS) and unfolded protein response (UPR) play vital roles in osteoarthritis by inducing oxidative stress, which activates endoplasmic reticulum (ER) stress and leads to mitochondrial injury to cause a cascade of rat chondrocyte apoptosis [3–6]. ER is a crucial tubular structure in chondrocytes on account of a mass of extracellular matrix (ECM) secreted by chondrocytes during the course of endochondral ossification [5]. Accumulation of ROS and UPR in the ER activates oxidative stress and ER stress, subsequently inducing the apoptosis of chondrocytes [7–9]. Accumulation of UPR is detected by three primary molecular sensors, including protein kinase RNA-like endoplasmic reticulum kinase (PERK), activating transcription factor 6 (ATF6), and inositol-requiring protein-1*α* (IRE1*α*) [10]. All three pathways can trigger ER stress-related apoptosis in oxidative stress-induced chondrocyte. In this study, we used tert-Butyl hydroperoxide (TBHP) which is more stable and long-lasting than H_2_O_2_ as an oxidative stress inducer. It has been extensively used for the research on the mechanism of osteoarthritis [11–13].

Silent information regulator factor 2-related enzyme 1 (SIRT1) is a NAD+-dependent class 3 histone deacetylase and is activated in response to cellular stress [14] and aging-related diseases [15, 16]. SIRT1 exerts antiapoptotic function through the deacetylation of apoptosis-inducible nonhistone proteins including P53 [17], forkhead family proteins [18], and RelA/p65 subunit of nuclear factor-*κ*B (NF-*κ*B) [19]. Previous studies verified the protective role of SIRT1 in different cells under oxidative stress and ER stress [20–23]. In osteoarthritis cartilage, research showed that the activation of SIRT1 is essential to the maintenance of articular cartilage homeostasis owing to its antiapoptotic effect [24, 25]. A potential mechanism of SIRT1-induced antiapoptosis effect in articular cartilage includes scavenging the free radicals and inhibiting oxidative stress [24, 26]. A recent study indicated that the activation of SIRT1 suppresses chondrocyte apoptosis through the inhibition of the PERK-eIF2*α*-CHOP axis of the UPR pathway [5, 27].

Curcumin is a polyphenolic compound which has a wide range of physiological properties including potent antioxidant [28], anti-inflammatory [29], and anticancer [30] effects. It is often used as a spice in our diets, and it has negligible side effects [28]. As a part of traditional Chinese medicine, curcumin is usually used for the treatment of different disorders such as cancers, asthma, diabetes, and many other diseases [31, 32]. Curcumin played an important role in the survival of various types of cells under oxidative stress [33–35]. Much of the useful effects were due to the significant antioxidant and radical scavenging properties of curcumin. Numerous previous in vitro and in vivo studies have showed that curcumin can alleviate chondrocyte apoptosis in human [36] and animals [37, 38]. Both oxidative stress and ER stress have been associated with the apoptosis of chondrocyte in OA. However, it remains obscure whether curcumin can protect chondrocytes through activating the protein level of SIRT1 and suppressing ER stress-induced apoptosis. In this study, we identified whether curcumin inhibited oxidative stress-induced rat chondrocyte apoptosis and explored the specific mechanisms. Moreover, we studied the therapeutic efficacy of curcumin in the anterior cruciate ligament transection (ACLT) rat OA model.

## 2. Materials and Methods

### 2.1. Reagents and Antibodies

Curcumin, tert-Butyl hydroperoxide solution (TBHP), thapsigargin (TG), DAPI, type II collagenase, and EX527 were purchased from Sigma-Aldrich (St. Louis, MO, USA). 0.25% trypsin was purchased from Gibco (NY, USA). Fetal bovine serum (FBS), Dulbecco's modified Eagle's medium- (DMEM-) F12 medium and PBS were obtained from HyClone (Logan, UT, USA). TUNEL staining kit was purchased from Beyotime (Jiangsu, China). The primary antibodies against SIRT1, cleaved caspase3, cleaved PARP, ATF4, GRP78, CHOP, PERK, p-PERK, eIF2*α*, p-eIF2*α*, and *β*-actin were purchased from Cell Signaling (Danvers, MA, USA); Bcl2 antibody was purchased from Abcam (Cambridge, UK). Cell counting kit-8 (CCK-8) was obtained from DOJINDO (Kumamoto, Japan).

### 2.2. Cell Isolation and Culture

The experimental procedures involving animal care and use were approved by the Animal Care and Use Committee of Shanghai Jiaotong University. All Sprague-Dawley (S-D) rats were maintained under specific pathogen-free (SPF) conditions. Primary chondrocytes were isolated from the bilateral hip joints of male 4-week-old rats which were purchased from Sino-British Sippr/BK Lab Animal Ltd. (Shanghai, China). Cartilage of rat hip joint was cut into 1 mm^3^ pieces in a sterile manner. Then, the cartilage pieces were digested with 0.25% trypsin for 1 h and incubated with 0.2% collagenase II in DMEM-F12 at 37°C and 5% CO_2_ for 6 h. After that, chondrocyte suspensions were centrifuged at 1200 rpm for 5 min and cultured at 37°C and 5% CO_2_ in DMEM-F12 complete culture medium which supplemented with 10% FBS and 1% penicillin and streptomycin. To avoid the loss of phenotype, rat chondrocytes no later than passage 2 were used for in vitro experiments.

### 2.3. Cell Proliferation Assay

The cytotoxicity of curcumin and TBHP on rat chondrocytes was examined by CCK-8 according to the manufacturer's protocol. Based on previous studies [12, 38], rat chondrocyte suspension was seeded into a 96-well plate with 5 × 10^3^ cells in each well and cultured for 24 h. Then, the chondrocytes were incubated with TBHP [12] (0, 5, 10, 20, 30, and 50 *μ*M/L) or curcumin [38] (0, 5, 10, 20, 25, and 50 *μ*M/L). Different concentrations of curcumin (0, 5, 10, 20, 25, and 50 *μ*M/L) were added into each well to treat rat chondrocytes for 2 h, followed by 20 *μ*M/L of TBHP treatment. Chondrocyte viability was assessed after 24 h, 48 h, and 72 h. Each well of the 96-well plate was added with 100 *μ*L of 10% CCK-8 solution and incubated for 2-4 h at 37°C. The optical density (OD) value at 450 nm of each well was detected and analyzed with a microplate reader.

### 2.4. Flow Cytometry Assay

Apoptotic degree of chondrocytes in each group was measured by using Annexin V Apoptosis Detection Kit (Sangon, Shanghai, China). After the treatment of TBHP and curcumin for 24 h, chondrocytes in each group was washed with PBS and trypsinized for 5 min in the absence of EDTA. The cells were obtained by centrifugation at 1200 rpm for 5 min and suspended in 200 *μ*L binding buffer. Then, 4 *μ*L Annexin V-FITC and PI were added into the flow tube and incubated for 10 min free of light [38]. The apoptotic rate (%) in each group was examined and analyzed using a flow cytometry (BD FACS ARIA II, SORP, USA).

### 2.5. Terminal Deoxynucleotidyl Transferase-Mediated dUTP Nick-End Labeling (TUNEL) Staining

TUNEL staining was used to detect the apoptotic levels in rat chondrocytes after different treatments for 24 h. After fixed with 4% PFA for 15 min, rat chondrocytes in different groups were rinsed with PBS for three times and permeabilized with 0.1% Triton-X 100 in PBS for 3 min. After that, apoptotic rat chondrocytes were stained by the usage of TUNEL staining kit and subsequently counterstained with DAPI for 10 min [38]. Images in different groups were observed by a confocal microscope. Then, we calculated the percentage of apoptotic chondrocytes and evaluated the apoptotic degree in each group.

### 2.6. Immunofluorescence Staining

The treated chondrocytes in each confocal dish were fixed with 1 mL 4% PFA for 25 min, permeated with 0.2% Triton X-100 in PBS for 5-10 min, and blocked with 5% BSA for 90 min at room temperature. Then, the primary antibodies against CHOP and SIRT1 were added to the plate and incubated for 24 h at 4°C. Subsequently, Alexa Fluor594- or Alexa Fluor488-conjugated second antibody was added at a 1 : 500 dilution in PBS and incubated free of light for 90 min. Ultimately, DAPI solution was added to each group and incubated for 10 min in the dark at ambient temperature. A confocal laser scanning microscope (Leica, IL, USA) was applied to detect and analyze the positive staining cells in each confocal dish, and the fluorescence intensity in each group was assessed by the ImageJ software 2.1.

### 2.7. Western Blot

To explore the function of curcumin on TBHP-induced chondrocyte apoptosis, the whole-cell protein was extracted by a lysis buffer which contains protease inhibitor cocktail (Roche, Stockholm, Sweden). After centrifugation at 12000 rpm for 15 min at 4°C, the supernatant in each group was collected. A BCA protein assay kit (Beyotime, Jiangsu, China) was used to measure the protein concentration of chondrocytes in each group. 20 *μ*g of total cellular protein in different groups was resolved by SDS-polyacrylamide gel electrophoresis and electrotransferred to poly-vinylidene difluoride (PVDF) membranes (Bio-Rad, Hercules, CA, USA). Following blocked with 5% BSA in Tris-buffered saline tween (TBST) at room temperature for 90 min, the bands were incubated overnight at 4°C with primary antibodies against the following proteins for western blot analysis: Bcl2 (1 : 200), cleaved caspase3 (1 : 100), cleaved PARP (1 : 100), SIRT1 (1 : 100), CHOP (1 : 100), GRP78 (1 : 100), ATF4 (1 : 100), PERK (1 : 100), p-PERK (1 : 100), eIF2*α* (1 : 100), p-eIF2*α* (1 : 100), and *β*-actin (1 : 250). Then, the bands were washed with TBST for three times and incubated with secondary antibodies for 90 min at ambient temperature. After that, the membranes were washed with TBST for three times; the Odyssey infrared imaging system (Li-Cor; Lincoln, NE, USA) was applied to detect and analyze the density of each band.

### 2.8. Real-Time PCR Analysis

Based on the manufacturer's instructions, total RNA of chondrocytes in each group were isolated using a RNA extraction kit (QIAGEN, Germany). 1000 ng of total RNA in each group was reverse transcribed to synthesize cDNA with the PrimeScript™ RT Master Mix (Takara, Japan). Then, the SYBR Premix Ex Taq™ (Takara, Japan), forward primer, and reverse primer of target gene were used in the process of cDNA amplification. A three-step PCR procedure of 5 s at 95°C, 20 s at 63.5° C, and 10 s at 72°C was used for 45 cycles; *β*-actin was used as a housekeeping gene [39]. The relative mRNA expression level of each target gene was calculated by using the 2^-ΔΔCT^ method [40]. The sequences of forward and reverse primer and the GenBank accession number of target genes were presented in Table 1.

### 2.9. OA Model

We purchased 40 male Sprague-Dawley (S-D) rats (8-week-old, weighing 300-350 g) from Sino-British Sippr/BK Lab Animal Ltd. (Shanghai, China). We performed arthrotomy without the transection of anterior cruciate ligament in the right knee joint of 10 S-D rats which were used as the Sham control group. Osteoarthritis was induced by surgical transection of the right anterior cruciate ligament (ACL) as previously described [41]. Then, the rats were randomly divided into four groups: Sham group, ACLT group, ACLT plus low-dose curcumin treatment group, and ACLT plus high-dose curcumin treatment group. Immediately after the ACLT surgery, curcumin was given once daily for 8 weeks by intraperitoneal injection [42, 43]. The low-dose curcumin (50 mg/kg) and the high-dose curcumin (150 mg/kg) were used in our study. After the treatment of curcumin for 8 weeks, we evaluated the changes of joint space and calcification on articular cartilage in each group by using the X-ray imaging.

### 2.10. Histological Analysis

Following 8 weeks of curcumin treatment, the right knee joint of rats in different groups was obtained after intraperitoneal injection of 10% chloral hydrate. The cartilage tissues in each group were fixed in 4% PFA for 24 h, and 10% EDTA was used to decalcify the specimens for 1 month. After that, the cartilage in each group was dehydrated, subsequently embedded in paraffin wax and cut into 5 *μ*m tissue sections [12]. Safranin O (S-O) staining and hematoxylin and eosin (H&E) staining were applied to evaluate the degeneration of articular cartilage in each knee joint, and the degradation change was assessed and analyzed by a Osteoarthritis Research Society International (OARSI) scoring system [44].

### 2.11. Immunohistochemical Analysis

Immunohistochemical staining was used to examine the levels of cleaved caspase3, SIRT1, and CHOP in different groups. The sections (5 *μ*m) were deparaffinised and rehydrated, followed by incubation with 3% H_2_O_2_ for 15 min and blocked with 5% BSA in TBST for 30 min. Then, primary antibody against CHOP, cleaved caspase3, SIRT1, and HRP-conjugated secondary antibody was used to incubate with the tissue sections in all groups [12]. Images in each group were captured by a light microscope. Then, we used the Image-Pro Plus software version 6.0 (Media Cybernetics, Rockville, MD, USA) to analyze the data in all groups. The level of cleaved caspase3, SIRT1, and CHOP in each group was examined and analyzed by the integral absorbance values.

### 2.12. Statistical Analysis

The experiments were performed at least three times. Our results were presented as mean ± standard deviation (SD). SPSS 13.0 software (SPSS Inc., Chicago, IL, USA) was used to analyze the data. Statistical analysis was performed by unpaired Student's *t* test for two groups and one-way ANOVA for more than two groups. The Kruskal–Wallis *H* test was applied to analyze the nonparametric data (OARSI scores). *P* value less than 0.05 was considered to indicate statistical significance.

## 3. Results

### 3.1. Effect of Various Concentrations of Curcumin on Chondrocyte Viability in the Presence or Absence of TBHP

**Figure 1:**
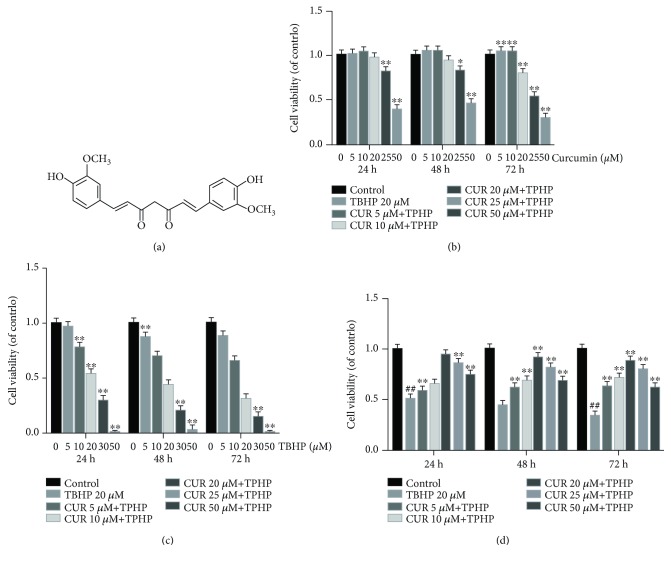
Effects of curcumin on the chondrocyte viability with or without TBHP. (a) Chemical structure of curcumin. (b, c) The cytotoxic effects of curcumin and TBHP on rat chondrocytes were examined at various concentrations for 24, 48, and 72 hours with a CCK8 kit. ^∗∗^ *P* < 0.01, ^∗^ *P* < 0.05 versus the control group. (d) The viability of TBHP-treated (20 *μ*M) chondrocytes after curcumin treatment. ^##^ *P* < 0.01 versus the control group; ^∗∗^ *P* < 0.01, ^∗^ *P* < 0.05 versus the TBHP treatment group. All values represent mean ± standard deviation (*n* = 5). TBHP: tert-Butyl hydroperoxide; CUR: curcumin.

The effect of curcumin on rat chondrocyte viability with or without TBHP was analyzed at different concentrations for 24, 48, and 72 h using the CCK-8 assay. We found that curcumin showed no cytotoxic effect on chondrocytes at concentrations of ≤20 *μ*M (Figure 1(b)). The results also revealed that 20 *μ*M TBHP reduced the viability of chondrocytes significantly (Figure 1(c)), while curcumin (≤20 *μ*M) markedly ameliorated the cytotoxic effect of TBHP in a dose-dependent manner (Figure 1(d)). OA is a chronic degenerative disease which is under persistent oxidative stress, so we used 20 *μ*M TBHP which is relatively mild to stimulate rat chondrocytes for 24 h to mimic oxidative stress of osteoarthritis in vitro. Thus, 20 *μ*M of curcumin and 20 *μ*M of TBHP were chosen in the following experiments ultimately.

### 3.2. Curcumin Protected Chondrocytes from Oxidative Stress-Induced Apoptosis

**Figure 2:**
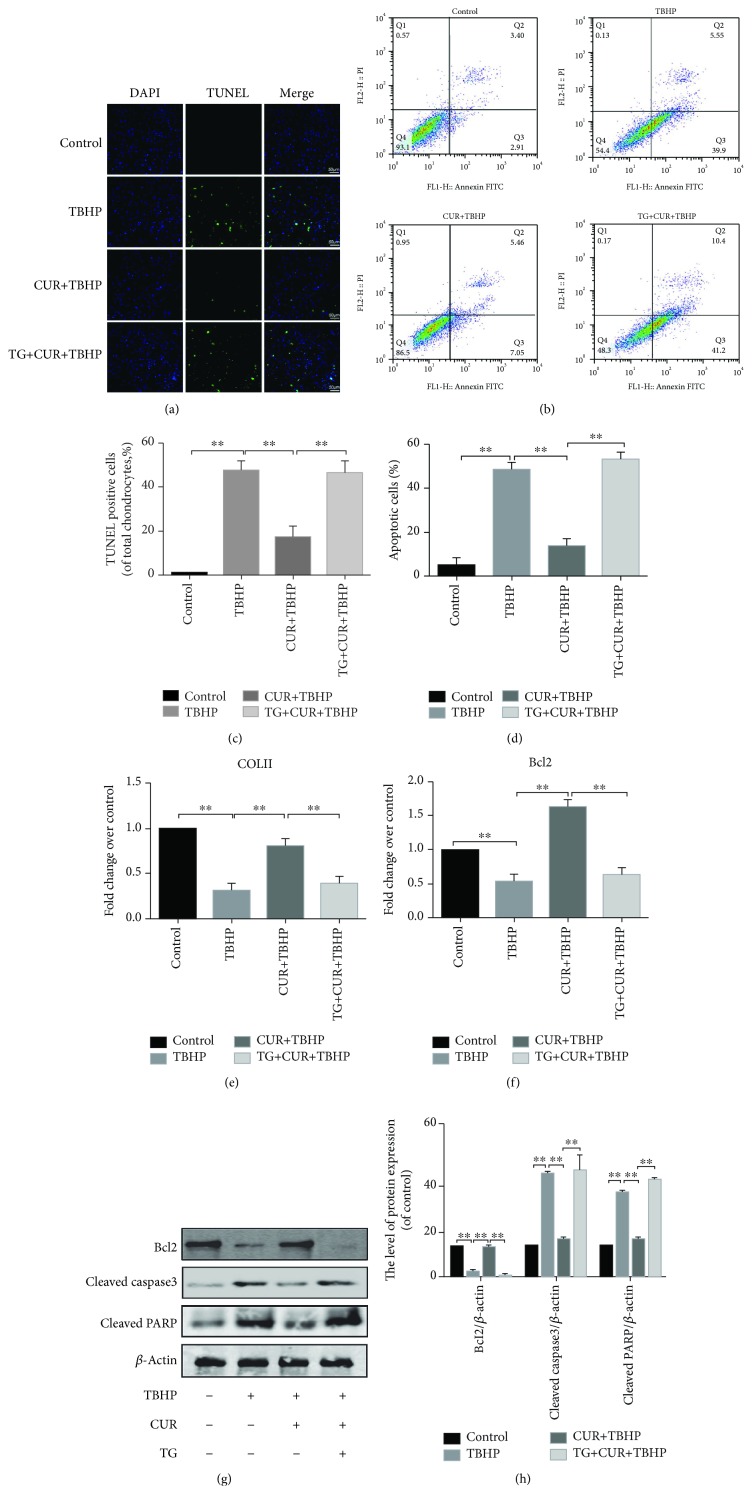
Curcumin inhibited chondrocyte apoptosis in TBHP-stimulated chondrocytes. (a, c) Apoptotic chondrocytes were examined using TUNEL fluorescence immunocytochemistry (green). Nuclei were counterstained with DAPI (blue) (bar: 50 *μ*m). (b, d) Flow cytometry was used to detect the apoptosis of chondrocytes which were labeled with Annexin V and PI fluorescence. (e, f) Real-time PCR analysis was used to examine the mRNA levels of collagen II and Bcl2 in each group. (g, h) The protein levels of Bcl2, cleaved PARP, and cleaved caspase3 in each group were detected. The values represent mean ± standard deviation (*n* = 5). ^∗∗^ *P* < 0.01. TUNEL: terminal deoxynucleotidyl transferase dUTP nick-end labeling; DAPI: 4′,6-diamidino-2-phenylindole; PI: propidium iodide; TBHP: tert-Butyl hydroperoxide; CUR: curcumin; TG: thapsigargin.

To test whether curcumin exerted an antiapoptotic effect on chondrocytes, we treated chondrocytes with 20 *μ*M TBHP, with or without curcumin. Rat chondrocytes were treated with 20 *μ*M TBHP for 2 h first, then treated with or without 20 *μ*M curcumin for another 24 h. As shown in Figure 2, curcumin treatment significantly decreased TBHP-induced apoptosis of chondrocytes. TUNEL staining and flow cytometry were used to detect the apoptotic degree of chondrocytes (Figures 2(a)–2(d)). Results of TUNEL staining showed that the apoptosis rate of chondrocytes stimulated with TBHP was markedly increased, and this effect was reversed by curcumin (Figures 2(a) and 2(c)). Meanwhile, the results of flow cytometry assay showed the same function of curcumin as TUNEL staining (Figures 2(b) and 2(d)). The real-time PCR analysis showed that curcumin significantly increased the expression of COLII and Bcl2, compared with TBHP-treated chondrocytes (Figures 2(e) and 2(f)). Moreover, the result of western blot demonstrated that curcumin downregulated the levels of proapoptotic proteins (cleaved caspase3 and cleaved PARP) and upregulated the Bcl2 expression level compared with the TBHP group (Figures 2(g) and 2(h)). These results indicated that curcumin may play a protective role in TBHP-induced chondrocyte apoptosis.

### 3.3. Curcumin Inhibited the ER Stress in TBHP-Treated Rat Chondrocytes

**Figure 3:**
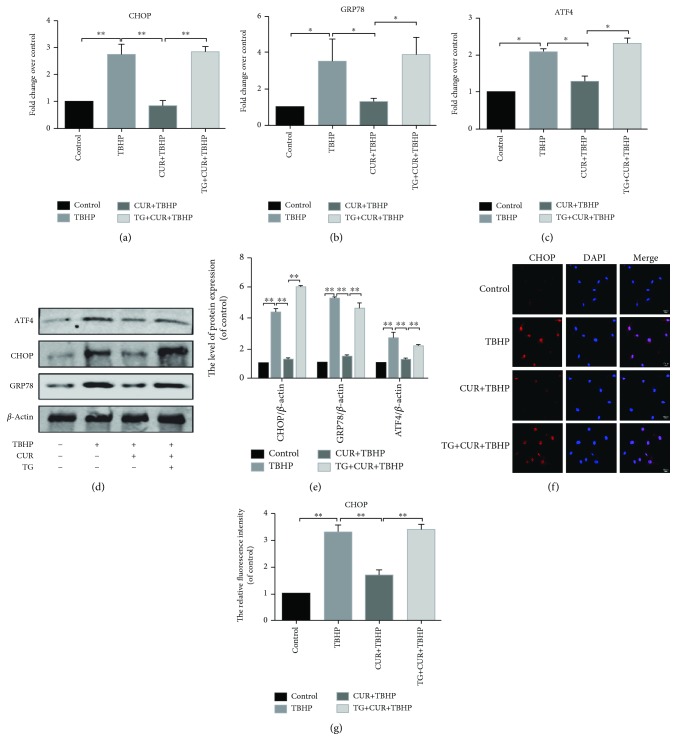
Curcumin inhibited ER stress in oxidative stress-induced rat chondrocytes. (a-c) The mRNA expression levels of CHOP, GRP78, and ATF4 in each group were examined by real-time PCR analysis. (d, e) Protein expression levels of CHOP, GRP78, and ATF4 were evaluated by western blot analysis. (f) CHOP immunofluorescence staining. Markedly increased red bright puncta indicated the upregulated expression of CHOP (bar: 10 *μ*m). (g) Quantitation of the number of CHOP positive chondrocytes in each group based on immunofluorescence staining. The results represent mean ± standard deviation (*n* = 5). ^∗^ *P* < 0 05, ^∗∗^ *P* < 0.01. TBHP: tert-Butyl hydroperoxide; CUR: curcumin; TG: thapsigargin.

To evaluate whether ER stress inhibition was related to the antiapoptotic effects of curcumin, ER stress-related biomarker CHOP, GRP78, and ATF4 were examined by real-time PCR (Figures 3(a)–3(c)) and western blot analysis (Figures 3(d) and 3(e)). CHOP, GRP78, and ATF4 were markedly increased in TBHP-stimulated chondrocytes but was partially reversed by curcumin treatment. The protein expression levels of CHOP, GRP78, and ATF4 remained unchanged compared to the control group when we treated chondrocytes with curcumin alone (Supplementary Figures S1(a) and S1(b)).The data of immunofluorescence staining of CHOP was consistent with the results of western blotting and real-time PCR (Figures 3(f) and 3(g)).

### 3.4. Curcumin Attenuated TBHP-Induced Chondrocyte Apoptosis by Inhibiting ER Stress

To further explore whether ER stress was related to the protective effect of curcumin in TBHP-treated chondrocytes, thapsigargin (TG) was applied to activate ER stress in rat chondrocytes. Results of real-time PCR (Figures 3(a)–3(c)) and western blot results (Figures 3(d) and 3(e)) indicated that the treatment of TG markedly increased the levels of CHOP, GRP78, and ATF4, compared to the group of CUR+TBHP. In addition, immunofluorescence staining of CHOP showed that TG promoted the activity of ER stress (Figures 3(f) and 3(g)). As above, curcumin protected chondrocytes from oxidative stress-induced apoptosis. To confirm whether TBHP-induced apoptosis is attenuated by curcumin-induced inhibition of ER stress, we activated ER stress by using TG and measured the levels of biomarkers of apoptosis, including Bcl2, cleaved caspase3, and cleaved PARP (Figures 2(e)–2(h)). Flow cytometry assay (Figures 2(b) and 2(d)) and TUNEL staining (Figures 2(a) and 2(c)) were also used to detect the apoptotic level after the TG treatment. These results showed that the antiapoptotic effect of curcumin was inhibited by TG. Therefore, curcumin attenuated oxidative stress-stimulated chondrocyte apoptosis by suppressing ER stress.

### 3.5. Curcumin Increased the SIRT1 Expression and Blocked the PERK-eIF2*α*-CHOP Pathway in TBHP-Treated Chondrocytes

**Figure 4:**
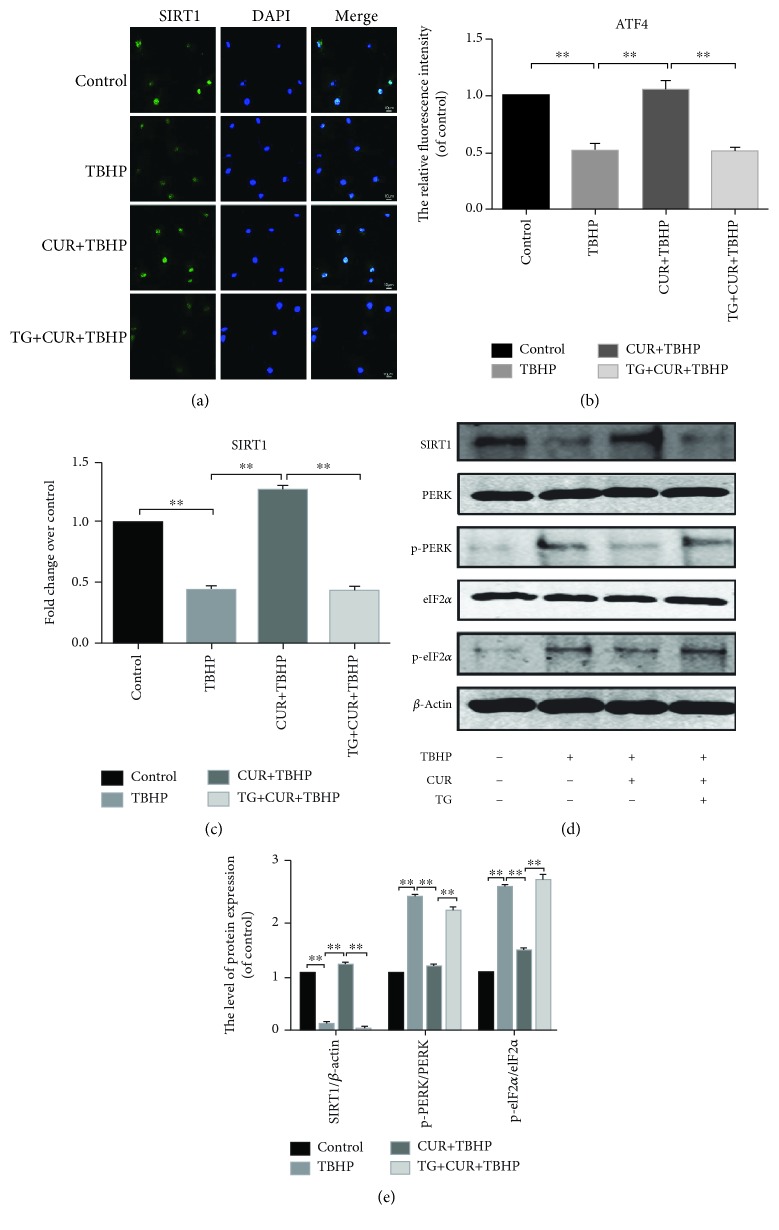
Curcumin promoted the expression of SIRT1 and inhibited the activation of the PERK-eIF2*α*-CHOP pathway. (a, b) Immunofluorescence staining of SIRT1 and quantitation of the number of chondrocytes positive for SIRT1 in different groups. Markedly increased green bright puncta indicated the upregulation of SIRT1 protein expression (bar: 10 *μ*m). (c) The mRNA expression levels of SIRT1 was assessed by real-time PCR analysis. (d, e) The protein expression levels of SIRT1 and PERK-eIF2*α*-CHOP pathway-related biomarkers were assayed by western blot analysis. All values represent mean ± standard deviation (*n* = 5). ^∗∗^ *P* < 0.01. TBHP: tert-Butyl hydroperoxide; CUR: curcumin; TG: thapsigargin.

Previous research indicated that the activation of SIRT1 suppressed chondrocyte apoptosis by the inhibition of the PERK-eIF2*α*-CHOP pathway [5]. Therefore, we detected the expression degrees of SIRT1 and the PERK-eIF2*α*-CHOP pathway-related proteins. As shown in Figure 4(d), TBHP decreased the expression of SIRT1 in rat chondrocytes while the treatment of curcumin abrogated the effect of TBHP. We also found that the protein expression level of SIRT1 was unchanged compared to the control group when we treated chondrocytes with curcumin alone (Supplementary Figures S1(a) and S1(b)). Moreover, our data showed that curcumin inhibited the upregulation of the protein expression of p-PERK and p-eIF2*α* in oxidative stress-induced chondrocytes (Figures 4(d) and 4(e)). The data of real-time PCR (Figure 4(c)) and immunofluorescence staining of SIRT1 (Figures 4(a) and 4(b)) were consistent with the results of western blotting.

### 3.6. Curcumin Suppressed ER Stress-Associated Apoptosis via Activation of SIRT1 and Inhibition of the PERK-eIF2*α*-CHOP Signaling Pathway in Chondrocytes

**Figure 5:**
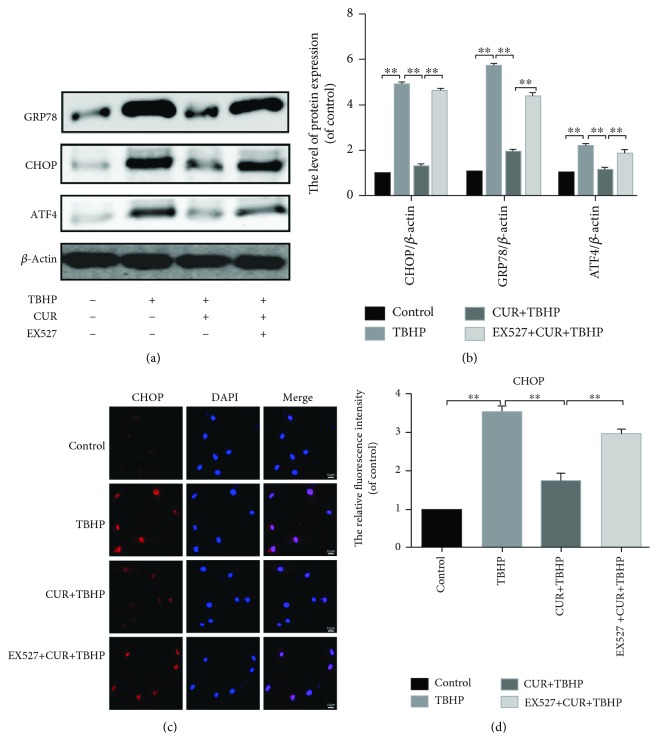
EX527 abrogated the inhibiting effect of curcumin on oxidative stress-induced ER stress in chondrocytes. (a, b) The protein expression levels of CHOP, GRP78, and ATF4 were evaluated by western blot analysis. (c, d) The representative CHOP was detected by the immunofluorescence counterstained with DAPI staining (bar: 10 *μ*m). The fluorescence intensity of CHOP was analyzed by ImageJ. All values represent mean ± standard deviation (*n* = 5). ^∗∗^ *P* < 0.01. TBHP: tert-Butyl hydroperoxide; CUR: curcumin; EX527: a classic SIRT1 inhibitor.

To evaluate the roles of SIRT1 and PERK activation in ER stress-induced apoptosis of chondrocytes, we activated ER stress by using TG. Our results showed the protein expression level of SIRT1 decreased and the protein expression levels of p-PERK and p-eIF2*α* upregulated in TG-stimulated rat chondrocytes (Figures 4(d) and 4(e)). Furthermore, we used a known SIRT1 inhibitor, EX527. Our results showed that the expression of CHOP, GRP78, and ATF4 was increased in the EX527+CUR+TBHP group (Figures 5(a) and 5(c)). These results indicated that the inhibition of SIRT1 and the activation of PERK were associated with ER stress-induced chondrocyte apoptosis.

### 3.7. Curcumin Inhibited the PERK-eIF2*α*-CHOP Axis of the ER Stress through the Activation of SIRT1 in TBHP-treated Chondrocytes

**Figure 6:**
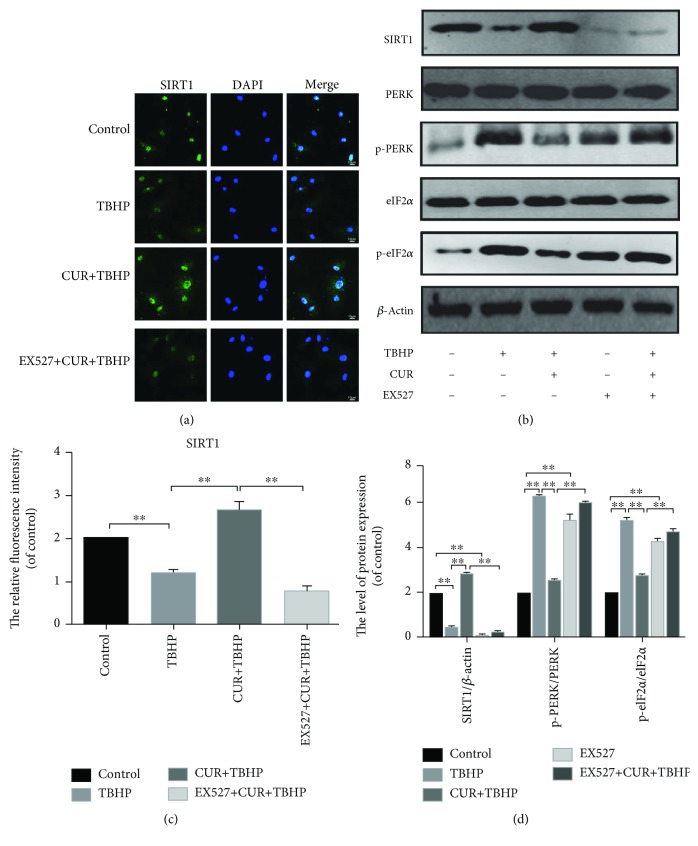
EX527 abrogated the protective of curcumin in TBHP-stimulated rat chondrocytes by inhibiting the PERK-eIF2*α*-CHOP pathway. (a, c) Immunofluorescence staining of SIRT1 (bar: 10 *μ*m). The fluorescence intensity of SIRT1 was analyzed by ImageJ. (b, d) The expression of SIRT1 and PERK-eIF2*α*-CHOP pathway-related proteins was assessed after the treatment of EX527 by western blotting analysis. All values represent mean ± standard deviation (*n* = 5). ^∗∗^ *P* < 0.01. TBHP: tert-Butyl hydroperoxide; CUR: curcumin; EX527: a classic SIRT1 inhibitor.

As described above, curcumin attenuated the oxidative stress-induced apoptosis of chondrocytes by activating the expression of SIRT1 and inhibiting the PERK-eIF2*α*-CHOP pathway. However, the relationship between the SIRT1 and the PERK-eIF2*α*-CHOP pathway is still unclear. Therefore, we used EX527 to inhibit the expression of SIRT1 in rat chondrocytes. After using EX527, the protein level of SIRT1 was decreased and the protein levels of p-PERK and p-eIF2*α* which decreased by curcumin were raised again in TBHP-treated chondrocytes (Figures 6(a) and 6(b)). Our data indicated that SIRT1 can decrease ER stress in chondrocytes through inhibiting the PERK-eIF2*α*-CHOP pathway.

### 3.8. Curcumin Ameliorated Chondrocyte Apoptosis and Inhibited ER Stress in Rat ACLT Model

**Figure 7:**
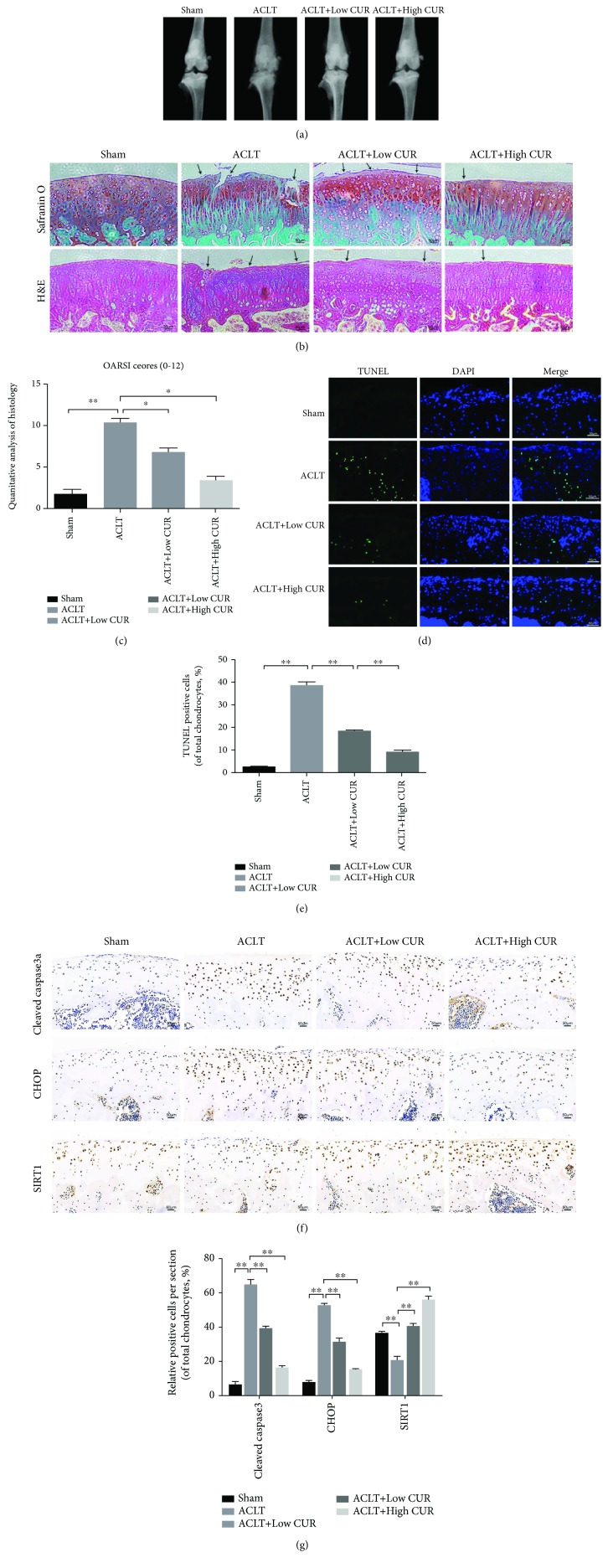
Curcumin inhibited apoptosis of chondrocytes and ER stress in the rat ACLT model. (a) The degenerative changes of articular cartilage in each group were assessed by X-ray imaging. The joint degenerative changes include the calcification of cartilage surface and the narrowing of knee joint space. (b) Histological analysis and microscopic observation of cartilage destruction in each group were evaluated at 8 weeks postsurgery by Safranin O staining and H&E staining (bar: 50 *μ*m). The defects and destruction of cartilage surface indicated the osteoarthritis pathological changes of rat knee joint. Lesions of articular cartilage were indicated by black arrows. (c) Osteoarthritis Research Society International (OARIS) scores of articular cartilage in four groups as indicated. (d, e) TUNEL staining of apoptotic chondrocytes and quantitation of TUNEL staining in each group (bar: 50 *μ*m). (f, g) IHC staining of cleaved caspase3, CHOP, and SIRT1 expression and the quantitation of the IHC staining in each group (bar: 50 *μ*m). All results represent mean ± standard deviation (*n* = 5). ^∗∗^ *P* < 0.01, ^∗^ *P* < 0.05. ACLT: anterior cruciate ligament transection; Low CUR: 50 mg/kg/day curcumin; High CUR: 150 mg/kg/day curcumin, IHC staining, immunohistochemical staining.

To explore the protective function of curcumin on OA in vivo, ACLT rat OA models were established surgically, followed by intraperitoneal injection of 50 mg/kg curcumin and 150 mg/kg curcumin once daily for 8 weeks. Our results of X-ray imaging demonstrated that the ACLT group showed sclerosis of cartilage surface and narrowing of the space of knee joint compared to the Sham group (Figure7(a)). However, this condition was reversed by the treatment of curcumin in a dose-dependent manner. The results of Safranin O (S-O) and H&E staining (Figure 7(b)) showed that the surface of cartilage in rat knee joint was smooth and stained red in the Sham operation group. In the group of ACLT, we found severe destruction, erosion and lesions of articular cartilage, remarkable reduction of chondrocytes, and enormous loss of proteoglycan. However, curcumin ameliorated the loss of chondrocytes and proteoglycan and inhibited the progression of OA in ACLT rats in a dose-dependent manner. Meanwhile, Osteoarthritis Research Society International (OARSI) scores presented a great consistent with histological analysis (Figure 7(c)). OARSI score of the group of ACLT was markedly increased compared with the Sham operation group, while the curcumin-treated group had markedly lower OARSI score than the ACLT group in a dose-dependent manner. TUNEL staining demonstrated that apoptotic chondrocytes in the group of ACLT were markedly increased compared to the Sham operation group. The treatment of curcumin decreased the apoptosis level in a dose-dependent manner (Figures 7(d) and 7(e)). Immunohistochemical staining demonstrated that curcumin significantly reduced the levels of cleaved caspase3 and CHOP in rat cartilage compared to the Sham group, consistent with the in vitro studies (Figures 7(f) and 7(g)). These results indicated that curcumin inhibited ER stress and had a significant antiapoptotic function in vivo, which were consistent with in vitro results.

## 4. Discussion

The development and progression of osteoarthritis have been attributed to the apoptosis of massive chondrocytes, which leads to the degeneration of cartilage and thickening of the subchondral bone. Moderate oxidative stress and ER stress are adaptive protective mechanism in chondrocytes. However, excessive oxidative stress and related ER stress play crucial roles in the apoptosis of chondrocytes in OA [6, 45]. Plenty of unfolded proteins which are induced by oxidative stress could promote the phosphorylation of ATF6 and PERK, leading to the activation of CHOP and several downstream apoptotic proteins [46]. Our results firstly demonstrated curcumin (20 *μ*M) markedly reduced apoptotic chondrocytes and the levels of apoptotic biomarkers including cleaved caspase3 and cleaved PARP, which were upregulated in TBHP-stimulated chondrocytes. Interestingly, we also found that curcumin still exerted a protective effect at 50 *μ*M in TBHP-treated (20 *μ*M) chondrocytes although it is cytotoxic at this concentration and need further investigations.

CHOP and GRP78 are canonical biomarkers for ER stress [47, 48]. Our results demonstrated that curcumin (20 *μ*M) markedly decreased the expression level of CHOP and GRP78, suggesting that curcumin suppressed oxidative stress-induced ER stress. In eukaryotic cells, ER stress is mediated by three branches of the unfolded protein response, including PERK, IRE1*α*, and ATF6 [7]. Previous studies indicated that the increased expression of phosphorylated PERK [49] and decreased expression of PERK [50, 51] promoted the degeneration of cartilage in OA and the inhibition of ER stress could attenuate the development of OA [12]. In the present study, we firstly found that curcumin could attenuate ER stress by suppressing the PERK-eIF2*α*-ATF4-CHOP pathway. To reveal the relationship between ER stress and chondrocyte apoptosis, we used TG, a classic ER stress inducer. We found the antiapoptotic effect of curcumin was reversed by TG treatment. This study demonstrated for the first time that the protective function of curcumin was associated with the inhibition of the PERK-eIF2*α*-ATF4-CHOP signaling pathway, which directly suppressed the expression of CHOP and subsequent apoptotic activation in chondrocytes. However, it remains unclear whether another two pathways of ER stress are related to the protective effect of curcumin in OA and need further studies.

Previous studies demonstrated that SIRT1 inhibited ER stress by scavenging the free radicals and inhibiting oxidative stress and could be an effective target for the treatment of OA [24–26]. Our recent study also showed that pharmacological activation of SIRT1 could inhibit chondrocyte apoptosis and cartilage degeneration by suppressing oxidative stress and ER stress [27]. Therefore, the fundamental purpose of our studies was to explore the potential role of curcumin as a regulator of SIRT1 in oxidative stress-induced chondrocytes and the specific mechanism by which it modulates oxidative stress-induced ER stress. Our results showed for the first time that the expression of SIRT1 was decreased by the treatment of TBHP while this function was reversed by curcumin. Moreover, we found that pretreatment with EX527 markedly increased the protein expression level of CHOP and GRP78 in TBHP-stimulated chondrocytes, suggesting that SIRT1 could inhibit the apoptosis of chondrocytes by attenuating ER stress.

In support of our results, previous related studies have shown that SIRT1 plays a crucial part in the regulation of ER stress-related apoptosis in different cells [20–23]. Moreover, SIRT1-deficient cells are sensitive to oxidative stress [52, 53], further proving the idea that SIRT1 plays an essential role in protecting against oxidative stress and related apoptosis. A recent research has shown that SIRT1 facilitated growth plate chondrogenesis and prevented chondrocyte apoptosis through the regulation of the PERK-eIF2a-CHOP axis of ER stress [5]. Our present study presented a great consistent with this research and firstly indicated that SIRT1 inhibition by EX527 increased the expression of ATF4 and the ratio of p-PERK/PERK and p-eIF2*α*/eIF2*α*. These results demonstrated that the important role of SIRT1 against ER stress-induced chondrocytes apoptosis is related to the inhibition of the PERK-eIF2*α*-ATF4-CHOP pathway. Taken together, our study is the first to suggest that curcumin could suppress the PERK-eIF2*α*-ATF4-CHOP pathway through promoting the expression of SIRT1 in oxidative stress-stimulated chondrocytes in vitro. Identically, it has the same problem that is whether another two pathways of ER stress are associated with the expression of SIRT1 and the protective effect of curcumin in OA and need further studies.

OA progression involves severe destruction and lesions of cartilage on the joint surface and chondrocyte apoptosis. The ACLT rat model is a classic OA model and was used in many studies [1, 41]. In the present study, we used the ACLT rat model to mimic OA progression. Liu et al. studied the protective effects of curcumin on lung injury after cardiopulmonary bypass (CPB) in a rat CPB model [54]. In their study, animals were pretreated with low-dose curcumin (50 mg/kg) and high-dose curcumin (200 mg/kg) with intraperitoneal injection in rats with CBP-induced lung oxidative damage [54]. Gaedeke et al. studied the effect of curcumin on glomerular fibrosis with a maximum does of 200 mg/kg body weight by intraperitoneal injection [55]. But the periods of curcumin injection in these above studies were relatively short (1 day or 6 days, respectively). In our study, we chose the maximum dose of 150 mg/kg to avoid the toxicity of curcumin which was caused by long-term injection (8 weeks). It has been reported that curcumin has a dose-dependent effect to ameliorate collagen-induced arthritis [56]. In our study, we used low dose (50 mg/kg) and high dose (150 mg/kg) to test whether curcumin slow osteoarthritis progression in the ACLT model in a dose-dependent manner. In the present study, we found that curcumin significantly attenuated knee joint degradation and decreased chondrocyte apoptosis in ACLT rats in a dose-dependent manner, which were consistent with previous studies [57, 58]. Previous studies showed that curcumin could inhibit the degradation of articular cartilage in vivo by suppressing inflammatory response [57] and activating autophagy [58]. However, our study demonstrated for the first time that curcumin could reduce the protein levels of cleaved caspase3 and CHOP and promoted the expression of SIRT1 in a dose-dependent manner, which indicated that activation of SIRT1 and inhibition of ER stress are potential mechanisms underlying the antiapoptotic function of curcumin in the ACLT rat OA model.

**Figure 8:**
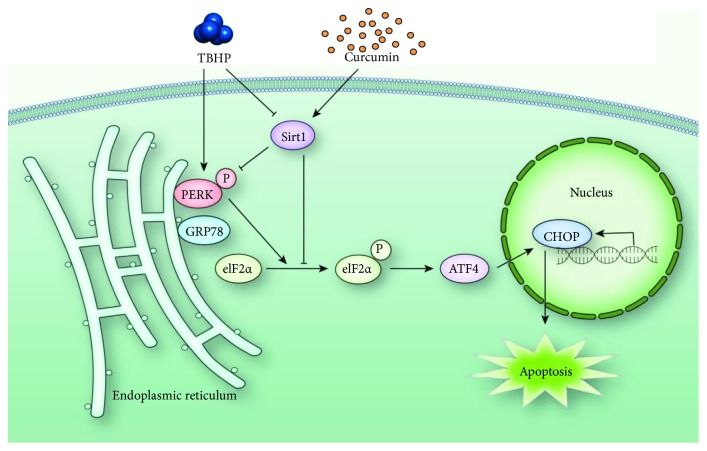
Schematic illustration of the mechanism of curcumin protective effects against TBHP-treated rat chondrocytes.

In conclusion, our present study firstly demonstrated that curcumin could ameliorate osteoarthritis progression by inhibiting ER stress-induced chondrocyte apoptosis in vitro and in vivo. The inhibition of ER stress and related PERK-eIF2*α*-ATF4-CHOP signaling pathway stimulated by curcumin protected rat chondrocytes against apoptosis by promoting the expression of SIRT1 (Figure 8). The therapeutic efficacy of curcumin was also confirmed in vivo. These results indicated the potential of curcumin as a treatment of osteoarthritis.

## Figures and Tables

**Table 1:** The primer sequences of target genes.

Gene	GenBank accession no.	Forward primer (5′-3′)	Reverse primer (5′-3′)
Collagen II	NM_012929.1	ACGCTCAAGTCGCTGAACAACC	ATCCAGTAGTCTCCGCTCTTCCAC
Bcl2	NM_016993.1	ACGGTGGTGGAGGAACTCTTCAG	GGTGTGCAGATGCCGGTTCAG
CHOP	NM_001109986.1	CTGAAGAGAACGAGCGGCTCAAG	GACAGGAGGTGATGCCAACAGTTC
GRP78	NM_013083.2	ACACCTGACCGACCGCTGAG	GCCAACCACCGTGCCTACATC
ATF4	NM_024403.2	GACCGAGATGAGCTTCCTGAACAG	CCGCCTTGTCGCTGGAGAAC
SIRT1	XM_006223877.1	GACGACGAGGGCGAGGAG	ACAGGAGGTTGTCTCGGTAGC
*β*-Actin	NM_031144	TCCCTGGAGAAGAGCTATGA	ATAGAGCCACCAATCCACAC

## Data Availability

The data used to support the findings of this study are available from the corresponding author upon request.

## Supplementary Materials

Supplementary Materials Figure S1: the effect of curcumin on SIRT1 and ER stress in chondrocytes under nonoxidative stress condition. (a, b) The protein expression levels of SIRT1 and ER stress biomarkers were detected by western blot and qualification analysis after the treatment of curcumin (20 *μ*M) with or without TBHP (20 *μ*M). All data represent mean ± S.D (*n* = 3). ^∗∗^ *P* < 0.01. CUR: curcumin; TBHP: tert-Butyl hydroperoxide.
