# Supplementary material for: Curcumin Inhibits the PERK-eIF2α-CHOP Pathway through Promoting SIRT1 Expression in Oxidative Stress-induced Rat Chondrocytes and Ameliorates Osteoarthritis Progression in a Rat Model
Source: Oxid Med Cell Longev. 2019 May 16;2019:8574386. doi: 10.1155/2019/8574386 (PMC6541984; doi:10.1155/2019/8574386)
Supplement: Supplementary Materials — Figure S1: the effect of curcumin on SIRT1 and ER stress in chondrocytes under nonoxidative stress condition. (a, b) The protein expression levels of SIRT1 and ER stress biomarkers were detected by western blot and qualification analysis after the treatment of curcumin (20 μM) with or without TBHP (20 μM). All data represent mean ± S.D (n = 3). ∗∗ P < 0.01. CUR: curcumin; TBHP: tert-Butyl hydroperoxide. [file 8574386.f1.pdf]

Supplementary Figure S1

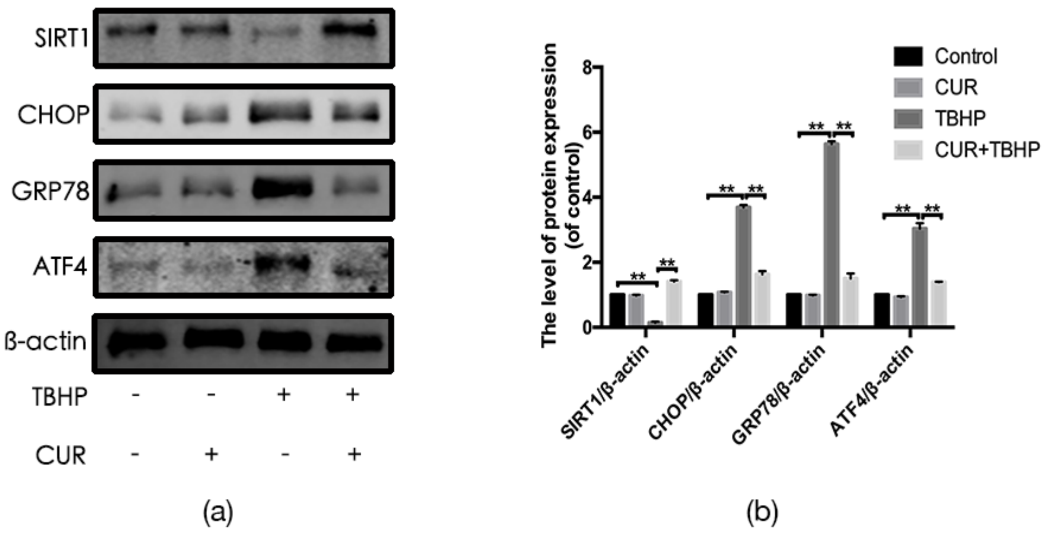

Figure S1: The effect of curcumin on SIRT1 and ER stress in chondrocytes under non-oxidative stress condition. (a, b) The protein expression levels of SIRT1 and ER stress biomarkers were detected by western blot and qualification analysis after the treatment of curcumin (20 μM) with or without TBHP (20 μM). All data represent mean ± S.D (n = 3). **P < 0.01. CUR, curcumin; TBHP, Tert-Butyl hydroperoxide.
